# Clinical and genetic analysis of multiple mitochondrial dysfunctions syndrome type 6 caused by biallelic *PMPCB* variants in children

**DOI:** 10.3389/fped.2026.1827306

**Published:** 2026-09-23

**Authors:** Yinyin Wu, Jihong Tang, Liya Zhang, Xiao Xiao, Bing Jia, Donghui Ge, Dangdang Zhang

**Affiliations:** Department of Neurology, Children’s Hospital of Soochow University, Suzhou, China

**Keywords:** children, gene, MMDS6, mutation, PMPCB

## Abstract

Multiple mitochondrial dysfunction syndrome 6 (MMDS6), caused by pathogenic variants in the *PMPCB* gene, is a rare autosomal recessive disorder. To date, only three studies describing a total of seven patients with MMDS6 have been published. In this report, we describe two siblings with MMDS6 who presented with significant motor and intellectual regression. Age of onset of the siblings was significantly later than that of previously reported cases. Whole-exome sequencing (WES) identified two novel compound heterozygous variants in *PMPCB* (c.1079C > G and c.1351C > T). These two variations originated from their father and their mother respectively. Although the two siblings have the same compound heterozygous mutation, their clinical symptoms show significant differences. Analysis via WB experiment indicated that these variants reduce *PMPCB* gene. PMPCB and Frataxin (FXN) protein expression. Furthermore, Analysis of protein stability prediction showed that the c.1079C > G variant decreases PMPCB protein stability. Based on these observation, we speculate that Both variants are candidate pathogenic variants pending functional validation. These findings expand the variant spectrum of *PMPCB*-related MMDS6 and revealed the potential pathogenic mechanism of *PMPCB* gene variants. We preliminary speculate that *PMPCB* might be a critical upstream regulator of FXN maturation.

## Introduction

Multiple mitochondrial dysfunction syndrome (MMDS) is a group of autosomal recessive mitochondrial disorders caused by mutations in nuclear genes, characterized by impaired energy metabolism, which leads to neurodevelopmental defects, muscle weakness, lactic acidosis, respiratory failure, and often early death. MMDS is associated with pathogenic variants in nine genes: *NFU1, BOLA3, IBA57, ISCA2, ISCA1, PMPCB, GCSH, FDX2*, and *FDXR* (1–4). While Mitochondrial proteins are encoded by both nuclear DNA (nDNA) and mitochondrial DNA (mtDNA), the majority are initially synthesized in the cytoplasm and subsequently imported into mitochondria via a complex transport system (5). Within mitochondria, protein presequences are cleaved by several processing peptidases, including Mitochondrial Processing Peptidase (MPP), inner membrane peptidase (IMP), mitochondrial intermediate peptidase (MIP), and the mitochondrial rhomboid protease Pcp1/PARL (5). Located in the mitochondrial matrix, MPP is a crucial enzyme that recognizes precursor proteins, facilitates their translocation across the mitochondrial membrane, and removes their N-terminal cleavable presequences. This processing is essential for proper protein folding and functional maturation. Dysfunction of MPP compromises the maturation of numerous mitochondrial precursor proteins, thereby impairing overall mitochondrial function and leading to severe, early-onset neurodegenerative diseases (6). Furthermore, short peptides generated by these proteases can accumulate within the organelle, exacerbating mitochondrial dysfunction. Consequently, the functional coupling between precursor protein processing and presequence degradation is vital for maintaining a stable organellar proteome (7). Structurally, MPP consists of an *α* and a *β* subunit; the latter, which harbors the enzyme's catalytic activity, is encoded by the *PMPCB* gene (8). First identified in 2018 (6), MMDS6 is an extremely rare condition with only six families reported to date. Consequently, additional cases with detailed clinical and experimental data are essential to elucidate its pathogenesis. The mutational spectrum of the *PMPCB* gene predominantly comprises missense variants that disrupt MPP function and drive disease progression. In this study, we report the clinical and molecular findings of two pediatric patients with motor and intellectual regression. Two compound heterozygous variants c.1351C > T (p.Arg451*) and c.1079C > G (p.Thr360Arg) of *PMPCB* were identified, which resulted in reduced expression of *PMPCB*, leading to the MMDS6 phenotype in these patients. Literatures describing patients with *PMPCB* -related MMDS6 were also collected and analyzed. We expanded the genotype spectrum of MMDS6 and provided deeper insights into the potential pathogenic mechanism of this disease.

## Materials and methods

### Patient and control

This study was approved by the Ethics Committee of Children's Hospital of Soochow University. A pair of siblings patients from the Department of Neurology of Children's Hospital of Soochow University was recruited and included in the subsequent studies. Additionally, peripheral blood samples from the brother of this siblings were selected as the experimental group(patient), while peripheral blood samples from a healthy 11-year-old male child were used as the control group(control). Both groups collected samples using two types of tubes: heparin tubes containing at least 4 ml of peripheral blood and PaxGene tubes containing at least 2 ml of peripheral blood. Western blot (WB) analysis was performed to detect the expression levels of PMPCB and FXN (frataxin) proteins in peripheral blood mononuclear cells across different samples.

### Genetic testing

Whole exome sequencing (WES) and copy number variation (CNV) were performed on DNA samples from the patients and their parents. Leukocyte DNA was extracted from 3 mL of peripheral blood from each core family member. Libraries were constructed and sequenced using an Illumina HiSeq sequencer (Illumina Inc., San Diego, CA, United States). For suspected variants, population and literature databases were used for annotation, including dbSNP, 1,000 Genomes, ExAC, Clinvar., GnomAD, OMIM, and HGMD. Interpretation of variants was performed according to ACMG guidelines (28). Candidate variants identified by WES were confirmed by Sanger sequencing.(Additional information on the WES in this study are listed in Supplementary Material).

### Conservative analysis

The human PMPCB protein and amino acid sequences of different species were retrieved from the Uniprot database, and the conservatism analysis was performed using the Align function provided by the database. Protein sequence conservation analysis was performed using UniProt Align (https://www.uniprot.org/align). PMPCB protein sequences from representative vertebrate and mammalian species were aligned.

### Quantitative polymerase chain reaction

Total RNA was extracted from the blood of the proband and the healthy control by PAXgene total RNA extraction kit (ZhiFan medicine, FG1101R-02, China). First-strand cDNA was reversed by HiScript II 1st Strand cDNA Synthesis Kit (Vazyme, R212-01, China). Wild type and variant fragments were amplified with *PMPCB*-F (5'- TGCACAGTTGGACTCTGGAT-3')and *PMPCB*-R (5’-TTCTTGGTGCCCTTGAAAGC-3'). *β*-Actin (internal control) was amplified with *β*-Actin-F (5'-CACCATTGGCAATGAGCGGTTC-3')and *β*-Actin-R (5'-AGGTCTTTGCGGATGTCCACGT-3'). Quantitative polymerase chain reaction (qPCR) was performed with AceQ qPCR SYBR Green Master Mix (Vazyme, Q111-02, China) according to the official guidelines. The relative expression of *PMPCB* (wild type and variant) was normalized to the expression of *β*-Actin. (Given that the collection of skin fibroblasts involves trauma and may result in scarring, which was not acceptable to parents, the peripheral blood of the children was used as the experimental sample in the *in vitro* experiments.).

### Western blot

Proteins were extracted from blood samples collected from the proband and the healthy control using RIPA lysis and extraction buffer (P0013B, Beyotime, Shanghai, China). Protein concentrations were determined using a bicinchoninic acid (BCA) assay kit (P0010, Beyotime, Shanghai, China). Total protein (10*μ*g/lane) was loaded onto SDS-PAGE and transferred to a polyvinylidene difluoride membrane. After blocking with 10% skim milk for 1.5 h, the membrane was incubated with the primary antibody at 4°C overnight. The primary antibodies used in this study were polyclonal anti- *PMPCB* (DF12305, Affinity Biosciences, Jiangsu, China).*β*-Actin Mouse Monoclonal Antibody (AF0003, Beyotime, Shanghai, China). and Anti-Frataxin (ab175402, Abcam, Britain). (The qPCR experiment of the *PMPCB* gene in blood or the WB experiment of PMPCB protein in the sample PBMC did not have biological replicates. The qPCR experiment of the *PMPCB* gene in blood and the WB experiment of PMPCB protein in the sample PBMC were conducted by three technical replicates and a t-test was performed. The data are presented as mean ± standard deviation (Mean ± SD). The FXN antibody experiments on 293T cell or patient's PBMC or healthy control PBMC did not have biological or technical replicates, and no gray-scale analysis or t-test was conducted).

### Protein stability prediction

Protein stability of wild type and mutant was predicted by the DynaMut (https://biosig.lab.uq.edu.au/dynamut/), using the wild-type PDB file of PMPCB and the mutant loci (Thr360Arg). DynaMut is a tool for analyzing dynamic mutations in proteins. It utilizes molecular dynamics simulations to model the structure and dynamic changes of proteins, allowing for more accurate predictions of the effects of mutations on protein structure and function. By leveraging extensive experimental data and computational methods, DynaMut offers highly precise predictions of mutation effects, which can guide clinical treatment and drug design. In addition to predicting the impact of mutations on protein structure and function, DynaMut can also analyze the role of mutations in disease development, providing crucial information for disease diagnosis and treatment. Therefore, we have chosen DynaMut as our predictive tool. DynaMut predictions are computational in nature and have not been experimentally validated for the variants reported here. These results should be interpreted as hypothesis-generating only.

### 3D Protein structure modeling

Molecular modeling analysis was performed to estimate the variant in protein structure. WT and variations in PMPCB protein were predicted using Chimera (https://saves.mbi.ucla.edu/). Chimera Viewer software was used to visualize the structures between WT and variation proteins.

### Literature review

We searched The Wan fang, Pu wei, CNKI, the PubMed and Online Catalog of Human Genes and Genetic Disorders (OMIM) for previously reported *PMPCB* -related MMDS6 cases using the *PMPCB* gene and MMDS6 as keywords. In the article selection process, we screened literature that met the criteria based on the research objectives and subjects. Articles that were not relevant to our research topic or did not meet the required methods were excluded to ensure the accuracy and reliability of the study. For quality assessment of the included studies, we conducted professional evaluations on each study, including assessment of study design, sample selection, and data analysis. We ensured the quality and credibility of the research, thereby enhancing the reliability and scientific validity of the study results. The reported *PMPCB* variants and their corresponding clinical phenotypes were summarized.

### Statistical analysis

The statistical analysis was performed using GraphPad Prism software (Version 9, GraphPad Software Inc., San Diego, CA, USA) and Origin Pro 2019. The data were presented as means ± standard error of the mean (SEM).

## Results

### Case presentation

This report describes two pediatric siblings (P1 and P2). The siblings were separately the first and the third child of their healthy non-consanguineous parents, and were born at normal weeks of gestation with normal Apgar scores and birth weight. No family history was apparently known. The siblings exhibited approximately normal growth and development prior to symptom onset. P1, a 11-year-and-5-month-old Chinese boy, was first admitted to our department in August 2020, for progressive motor and cognitive decline over the preceding year. Clinical manifestations included an abnormal gait characterized by dragging of the left lower limb, reduced walking speed, and an inability to run or jump. He required handrail support to ascend or descend stairs. Cognitive and language functions also progressively deteriorated, previously he was able to recite texts, subsequently he lost the ability to read or construct sentences exceeding three to four words. His academic performance deteriorated significantly, with examination scores decreasing from approximately 80% to 10%–20%.P2, P1's sister and 7 years younger, developed an abnormal gait at approximately five years old, characterized by adduction and internal rotation of the left lower limb and a tiptoe gait. Her motor regression was progressive and severe; in contrast, the decline in her cognitive and language abilities was relatively mild. On admission, P1's physical examination revealed dysmetria on the finger-to-nose test, an unsteady gait, left-sided toe-walking, and left foot varus. Bony prominences were palpable on the lateral aspects of both feet. Left lower limb muscle strength was graded 4+/5, accompanied by mildly increased muscle tone. He exhibited pronounced left hindfoot varus that worsened with weight-bearing, as well as pes cavus. Ankle dorsiflexion was limited to approximately 10° with the knee extended but reached a neutral position with the knee flexed. Mild Achilles tendon tightness was noted. Ankle clonus was negative, patellar reflexes were diminished, and jumping ability was impaired. Physical examination of P2 revealed intact fine motor skills. Her left lower limb exhibited adduction, internal rotation, and hypertonia. The left patellar reflex was hyperactive, and the left Babinski sign was positive. Additionally, she exhibited reduced ankle dorsiflexion and a mild left ankle contracture. A comprehensive laboratory panel for P1—including peripheral blood analyses (blood gas, coagulation profile, erythrocyte sedimentation rate, cardiac enzymes, procalcitonin, ceruloplasmin, humoral immunity, blood ammonia, comprehensive thyroid function, autoantibodies, and tuberculosis antibodies)were all within normal limits. Cerebrospinal fluid (CSF) analysis and routine electroencephalography (EEG) were unremarkable. However, cranial magnetic resonance imaging (MRI) revealed cerebellar atrophy (Figure 1A), whereas a whole-spine MRI was unremarkable. For P2, peripheral blood tests, including lactate levels, were normal. Her cranial MRI also demonstrated cerebellar atrophy (Figure 1B). Further radiographic imaging of P2, including plain radiographs of the pelvis, lower limbs, and left ankle, alongside a whole-spine MRI, revealed no significant abnormalities. P1 was treated with oxiracetam alongside intravenous and oral B-complex vitamins. Upon discharge, his abnormal gait and language regression persisted, and post-discharge rehabilitation was recommended. At the most recent follow-up, P1 (age >15 years) ambulated with a forward-leaning posture and dragging left lower limb, though he retained the ability to ride a bicycle and ascend stairs with handrail support. Following an influenza A infection in November 2023, his condition deteriorated, marked by the new onset of neck stiffness, dysarthria, and tongue rigidity, rendering him nearly non-communicative. A recent intelligence quotient (IQ) assessment yielded a score of 52. P2 (currently age >8 years) presented with a significant decline in motor function, characterized by left lower limb dystonia. Symptoms are markedly exacerbated by emotional stress and exhibit distinct diurnal fluctuation, presenting milder in the morning and worsening by evening. In August 2022, P2 was diagnosed with dystonia at an outside institution and commenced treatment with levodopa/carbidop. However this therapy was discontinued after one month due to progressive symptom exacerbation. Despite ongoing rehabilitation, her motor function fluctuated. After being infected influenza A in November 2023, P2's condition deteriorated significantly. she cannot walk nor sit, just predominantly bedridden, lost the ability to write. Her cognitive function remains relatively stable with a recent IQ 85, though her speech is characterized by pauses during the articulation of long sentences. In 2025, P2 was admitted to the neurology department and pediatric intensive care unit (PICU)multiple times due to “limb tremors”, and diagnosed with dystonia. In January 2025, a re-examination of the cranial MRI showed that the cerebellar atrophy was similar to before. Currently, both siblings receive oral supplementation comprising coenzyme Q10, lysine, B-complex vitamins, and vitamins C and E. Additionally, P2 is treated with oral trihexyphenidyl, baclofen, and clonazepam.

**Figure 1:**
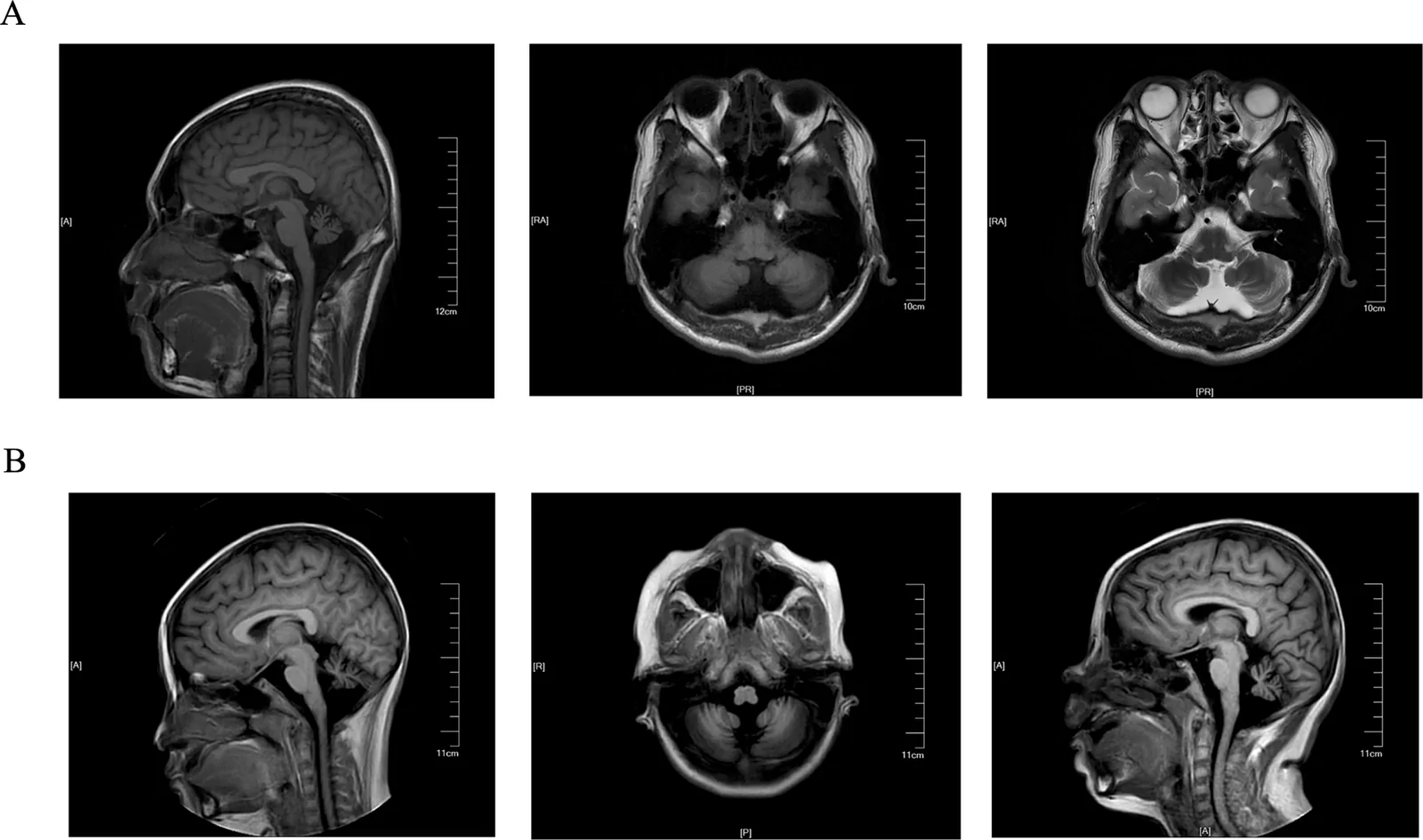
**(A)** cranial MRI of P1 showed cerebellar atrophy; (sagittal T1WI, axial T1WI, and T2WI, respectively). **(B)** cranial MRI of P2 showed cerebellar atrophy (June 2022: sagittal T1WI and axial T1WI. January 2025 sagittal T1WI, respectively).

### Genetic analysis

WES and CNV were performed to identify causative variants. Two novel compound heterozygous variants c.1079C > G (p.Thr360Arg) and c.1351C > T (p.Arg451*) of *PMPCB* gene were identified, which was inherited from their father and mother, respectively (Figure 2).

**Figure 2:**
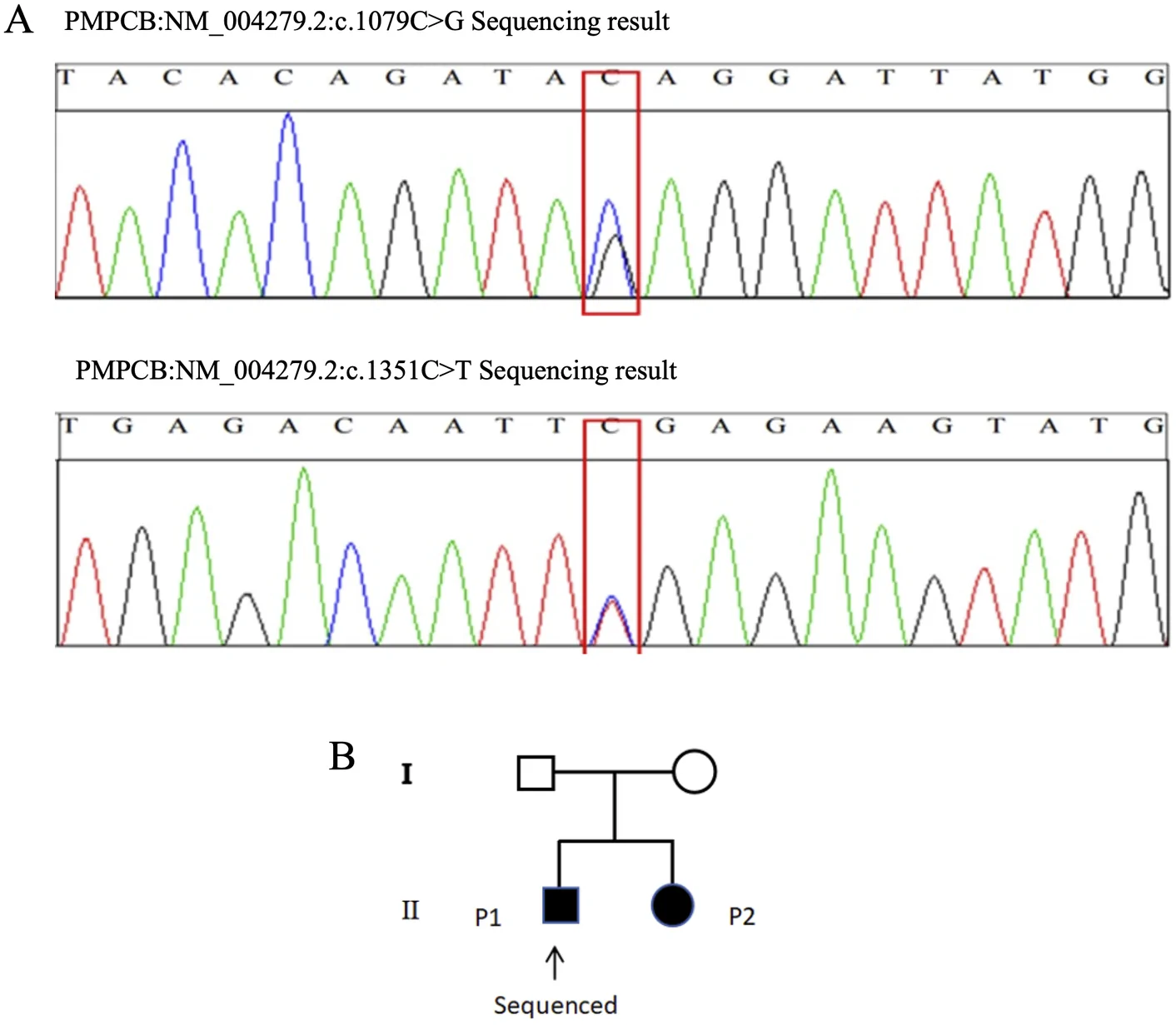
**(A)** sanger sequencing validation of compound heterozygous variants in gene PMPCB identified by trio-WES, red boxes mark the variant positions. **(B)** drawing of the pedigree, black symbols designate affected individuals.

Neither variant has been previously recorded in the Human Gene Mutation Database (HGMD). According to the Genome Aggregation Database (gnomAD), the allele frequency of each variant in the general population is less than 0.01%. According to the American College of Medical Genetics and Genomics (ACMG) guidelines, both variants were classified as variants of uncertain significance (VUS) (PM2_Supporting and PP3). The paternal variant c.1079C > G was predicted to be deleterious by 14 of 22 *in silico* prediction tools, of uncertain significance by four, and tolerated by four, yielding a REVEL score of 0.736 (Varsome data) (PP3). The maternal variant c.1351C > T is located in exon 12 (out of 13) of the *PMPCB* transcript, which encodes a 490-amino-acid protein. Its functional impact on the protein remains unknown, and no loss-of-function variants have been previously reported for this gene. Sanger sequencing confirmed the existence of each mutation. The homology analysis showed that the PMPCB proteins Thr360 was conservative among different species (Figure 3).

**Figure 3:**
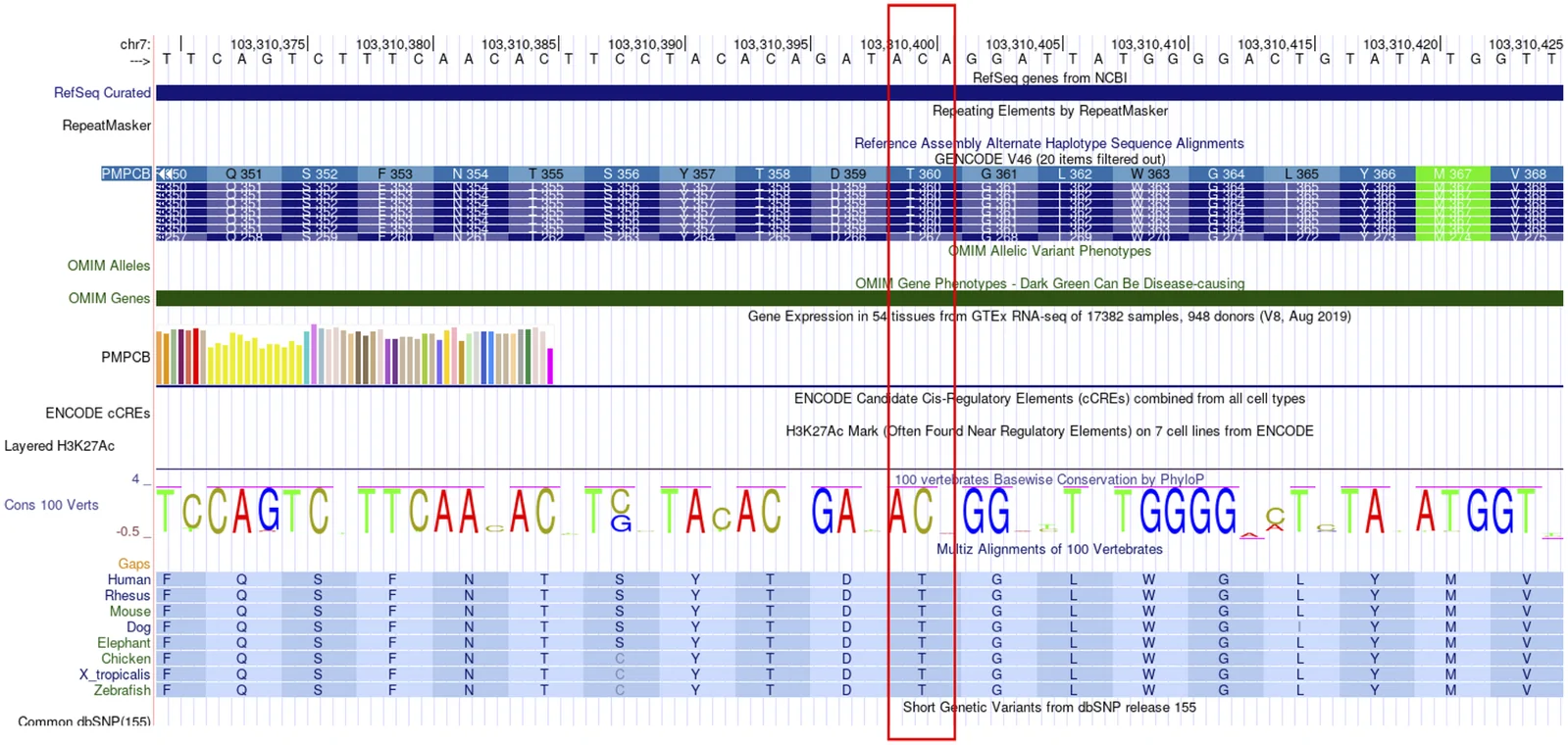
Species conservation analysis of the PMPCB protein. Multiple sequence alignment of PMPCB protein sequences across different species was performed using UniProt Align. The amino acid sequence surrounding codon 360 is shown for representative species. Thr360 is highlighted in red and is fully conserved across all species analyzed.

### Protein modeling

The protein model was constructed using AlphaFold (https://alphafold.ebi.ac.uk/), and its three-dimensional structure was visualized with Chimera (https://saves.mbi.ucla.edu/). Figure 4A illustrated the predicted tertiary structure of the wild-type PMPCB protein. In the wild-type structure, the threonine residue at position 360 (Thr360) forms hydrogen bonds (indicated by blue dashed lines) with Glu301, Tyr202, and Tyr357. Conversely, in the Thr360Arg mutant (Figure 4B), the substituted arginine residue maintains a hydrogen bond (red dashed line) with Tyr357, but losed the hydrogen bonds with Glu301 and Tyr202. Furthermore, thermodynamic stability predictions generated via the DynaMut (https://biosig.lab.uq.edu.au/dynamut/) indicated that the p.Thr360Arg mutation may reduce van der Waals interactions (blue dashed lines) between adjacent residues (Figures 4C,D). These computational predictions suggest that the p.Thr360Arg substitution may compromise protein stability; however, experimental validation (e.g., thermal stability assays, circular dichroism) is required to confirm these effects.

**Figure 4:**
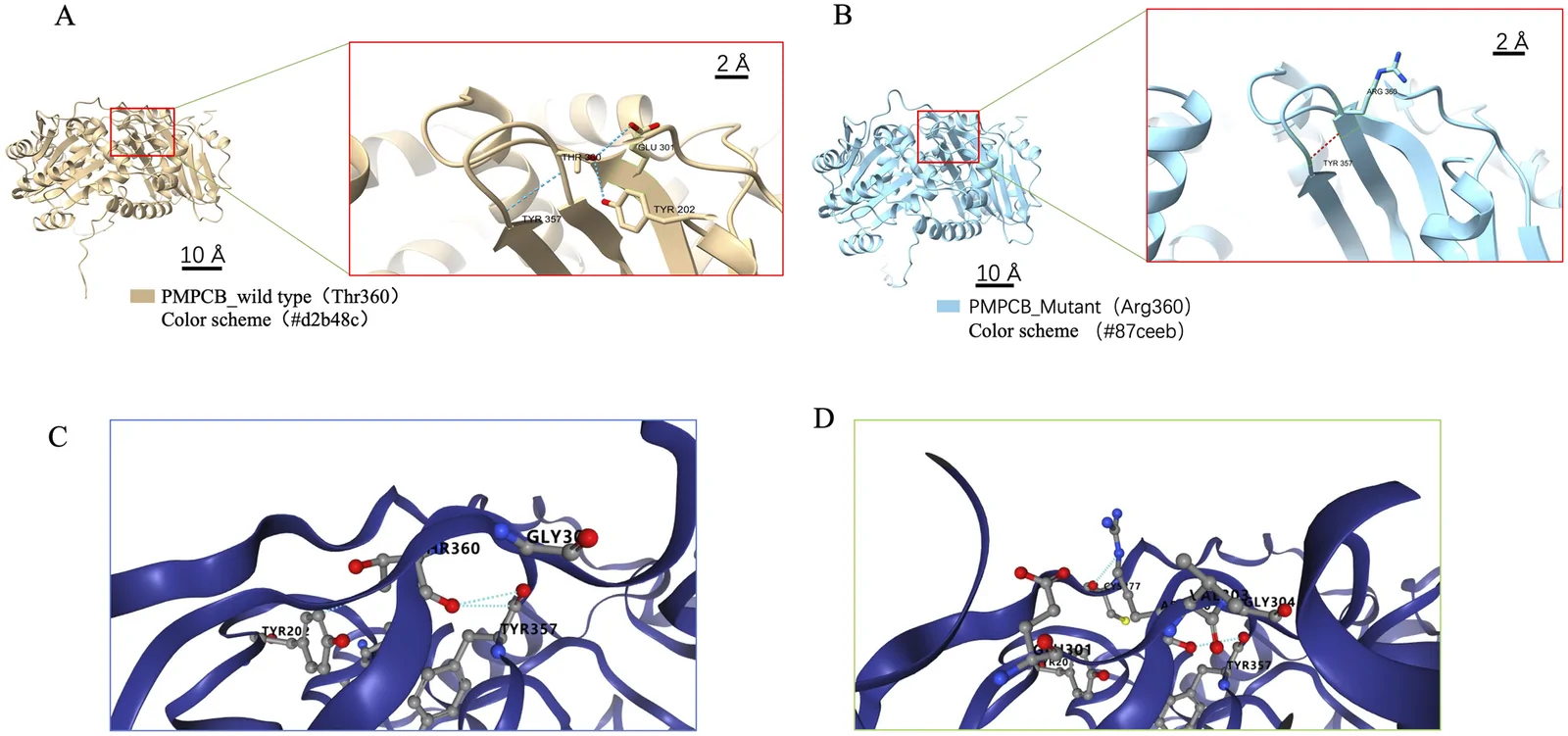
**(A)** shows the predicted tertiary structure of the wild-type *PMPCB* protein. **(B)** shows the predicted tertiary structure of the mutant protein of *PMPCB*. The prediction of the thermodynamic stability of the proteins using the DynaMut database and multi-analysis data tools suggests that *PMPCB* variant c.1079 C > G (p.Thr360Arg) caused a decrease in the van der Waals forces (blue dashed lines) between protein residues upon mutation. **(C)** WT protein. **(D)** Variant protein.

### Validation of the pathogenic variants

To investigate the effects of the two variants identified in the patient, we performed qPCR and western blot. Significant changes were observed in both assays. The mRNA expression of the *PMPCB* gene in the patient's blood was significantly decreased (1.00 ± 0.07 vs. 0.42 ± 0.05) (Figure 5A). Similarly, the protein expression of PMPCB was also significantly reduced in the patient's peripheral blood mononuclear cells (PBMCs) (1.06 ± 0.13 vs. 0.09 ± 0.04) (Figure 5B). Using an anti-FXN antibody (Abcam, ab175402), no band was detected in protein samples extracted from PBMCs, whereas a corresponding band was observed in samples from 293 T cells and the control (Figures 5C,D). These findings indicate that FXN expression in PBMCs was below the detection threshold. Based on these results, we can conclude that these mutations may affect the function of the PMPCB protein by influencing its quantity and stability. However, these findings are preliminary observations that still require independent validation. further functional analysis is still required to comprehensively understand the mechanisms by which these mutations impact protein function.

**Figure 5:**
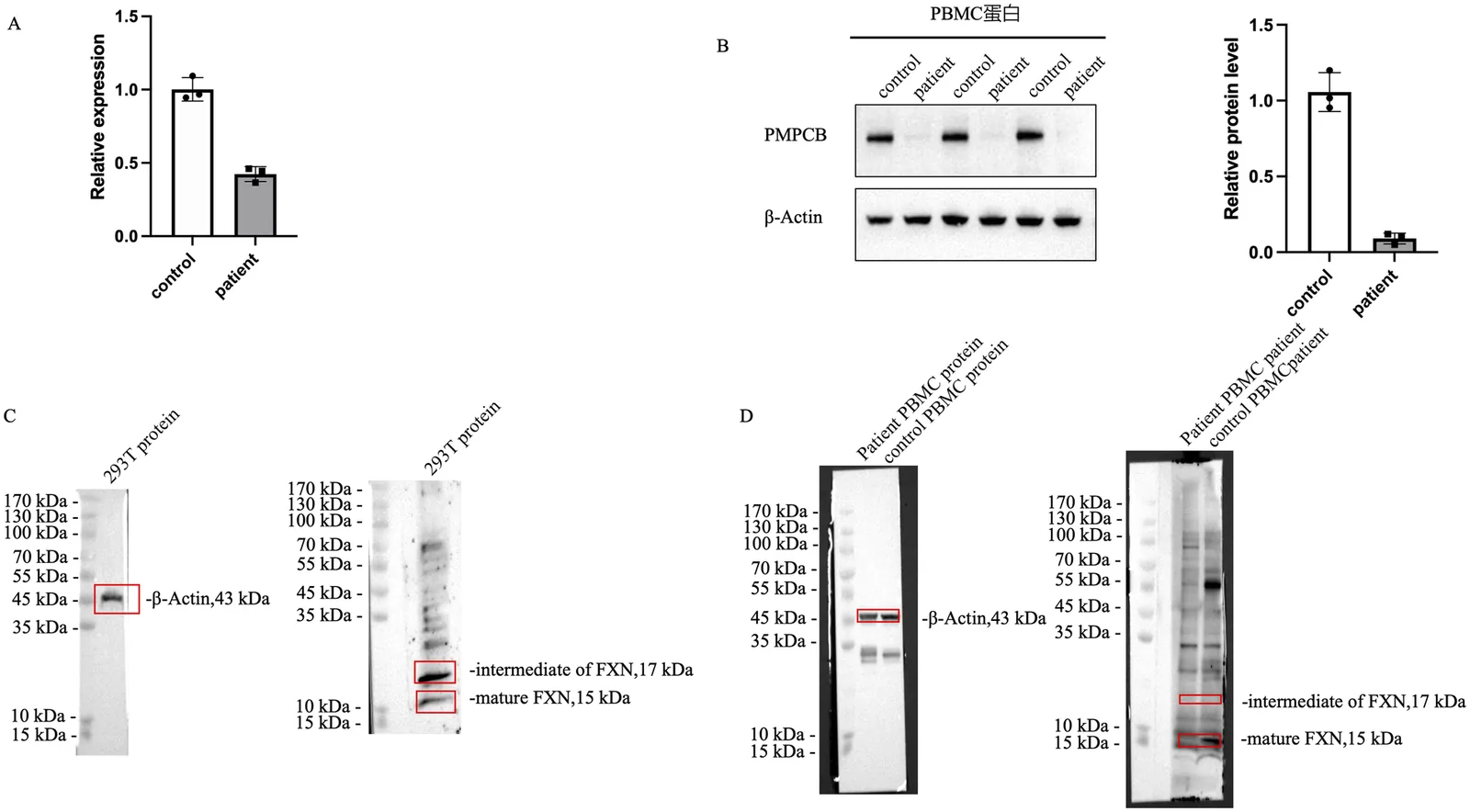
**(A)** relative expression level of the *PMPCB* gene in blood samples (with *β*-actin as the internal reference gene). **(B)** Relative expression level of the *PMPCB* protein in PBMCs (with *β*-Actin as the internal control). **(C)** Western blot results of FXN protein in 293T cell samples. **(D)** Western blot results of FXN protein in PBMC samples from the pediatric patient and the control.

### Summary of reported *PMPCB* -related MMDS6 cases

The Wan fang, Pu wei, and CNKI, as well as The OMIM and PubMed databases were searched for literatures related to “*PMPCB*” and “MMDS6” included up to September 2025. approximately 12 papers were related to the human *PMPCB* gene. After eliminating duplicate cases and those with incomplete data, a total of 3 publications involving 7 patients with *PMPCB*-related MMDS6 were reported. Seven *PMPCB* variants were identified in these 7 patients, all missense variants. and the children with relatively comprehensive clinical data had neurological manifestations. and all patients had MRI abnormalities. Combined with the two siblings in this article, there were a total of 9 cases. Detailed clinical features of the reported patients were shown in Table 1.

**Table 1:** Clinical characteristics of 9 pediatric cases with *PMPCB* gene variants.

Patient(Pn)	Age of onset (Gender)	Developmental history prior to onset.	Mode of Onset and Disease Progression	Blood lactate	Brain MRI	*PMPCB* gene	Sources of Genetic Variation	ACMG Pathogenicity assessment	Follow-up to the time of publication (status/age).
P1	10y(M)	Gross motor developmental delay	Abnormal gait with progressive regression of motor, language, and cognitive functions	Normal	Cerebellar atrophy	c.1079C > G	Parents	VUS	17y, Significant decline in verbal intelligence, while motor function remains relatively stable.
						c.1351C > T			
P2	5y(F)	Gross motor developmental delay	Loss of motor function, can only lie down	Slightly elevated	Cerebellar atrophy	c.1079C > G	Parents	VUS	10y, Marked motor regression.
						c.1351C > T			
P3 (6)	6 m(M)	Not reported in source publication	Epilepsy, inability to walk, language impairment, severe ataxia and dystonia, and poor weight gain	Slightly elevated	Progressive cerebellar atrophy with signal abnormalities, and abnormal signal intensity in the basal ganglia.	c.523C > T	Not reported in source publication	P	5y
						c.601G > C			
P4 (6)	12 m (F)	Not reported in source publication	Epilepsy, inability to walk, language impairment	Slightly elevated	Cerebellar atrophy, basal ganglia signal abnormality.	c.523C > T	Not reported in source publication	P	6y 1m
						c.601G > C			
P5 (6)	10 m(F)	Not reported in source publication	Inability to walk, language impairment, optic nerve atrophy, severe dystonia, poor weight gain	Slightly elevated	Cerebellar and brainstem atrophy, and abnormal signals in the basal ganglia and cerebral white matter	c.524G > A	Parents	P	Death (5y 6 m)
						c.530T > G			
P6 (6)	4 m(F)	Not reported in source publication	Epilepsy, inability to walk, language impairment, refractory epileptic encephalopathy	Normal	Progressive diffuse cerebellar atrophy with basal ganglia signal abnormality	c.1265T > C Homozygote	Parents	P	Death (6y)
P7 (6)	7 m(M)	Not reported in source publication	Epilepsy, inability to walk, language impairment, refractory epileptic encephalopathy	Normal	Progressive diffuse cerebellar atrophy with basal ganglia signal abnormality	c.1265T > C Homozygote	Parents	P	Death (5y5 m)
P8 (11)	3 m(M)	lift her head at three months old	Vomiting, feeding difficulties, slow weight gain, decreased muscle strength, and pulmonary hypertension	Slightly elevated	No abnormalities were observed at the initial stage, and no follow-up examination was performed subsequently	c.524G > A Homozygote	Parents	P	Death (6 m)
P9 (12)	2y5 m(F)	Motor and language development normal; walk at 14 months	Developmental regression, ataxia, aphasia, loss of walking ability at 6 years of age, and onset of migraines and severe gastrointestinal symptoms (vomiting) at 28 years of age.	Not reported in source publication	Basal ganglia and cerebellar atrophy	c.523C > T	Parents	P	Death (39y)
						c.1330-2A > Tp.(?)			

EEG, Electroencephalogram; MRI, Magnetic Resonance Imaging; y, Year; m, Month; M, Male; F, Female; ACMG, American College of Medical Genetics and Genomics; AD, Autosomal dominant inheritance; AR, Autosomal recessive inheritance; XD, Sex chromosome dominant inheritance; XL, Sex chromosome-linked inheritance; P, Pathogenic; LP, Likely pathogenic; VUS, Variant of Uncertain Significance; P1 and P2 are an older brother and a younger sister; P6 and P7 are an older sister and a younger brother.

## Discussion

Mitochondrial diseases comprise a group of rare metabolic disorders caused by mitochondrial dysfunction, with several hundred types identified to date. Among these, multiple mitochondrial dysfunctions syndrome (MMDS) represents a specific subtype (6). Notably, MMDS6 is caused by mutations in the *PMPCB* gene. This gene encodes the *β*-subunit of the MPP, which serves as the enzyme's catalytic core (5). Located on chromosome 7q22.1, *PMPCB* consists of 13 exons and encodes a ubiquitously expressed 490-amino acid protein. The critical function of *PMPCB* is highlighted by zebrafish studies in which the gene knockout proves lethal. In F1 offspring, this knockout induces morphological and central nervous system (CNS) defects similar to those caused by copper exposure. Conversely, ectopic *PMPCB* expression enhances the transcription of CNS-related genes during embryonic development in fish and mitigates the neurotoxic effects of copper. These findings underscore the crucial role of *PMPCB* in nervous system development across species, including humans (9). MMDS6 was linked to the dysfunction of the MPP catalytic subunit and the subsequent impairment of iron-sulfur clusters (ISCs) biogenesis (6).ISCs are essential cofactors for numerous proteins critical to mitochondrial energy production, including components of the electron transport chain (Complexes I, II, and III), pyruvate dehydrogenase, and *α*-ketoglutarate dehydrogenase (10), as well as the glycine cleavage system (11).Consequently, *PMPCB* mutations reduce the expression of the encoded protein, diminishing ISCs synthesis. This cascade impairs multiple critical metabolic pathways, including cellular respiration, leading to severe outcomes such as fatal lactic acidosis and defective glycine cleavage. Clinically, these metabolic defects manifest as severe neurological impairments, including encephalopathy, leukodystrophy, and psychomotor developmental delay (11).

Five cases of multiple mitochondrial dysfunctions syndrome 6 (MMDS6) caused by *PMPCB* gene mutations across four families were first reported (6). Subsequently, individual case of MMDS6 were reported in China (11) and the United Kingdom (12). In the case report section, we detail the clinical manifestations and treatment course of two Chinese siblings. Despite extensive clinical evaluation and management, their conditions remained poorly controlled, with persistent developmental regression. Following an influenza A infection in November 2023, the siblings exhibited pronounced motor and cognitive regression, resulting in further clinical deterioration. Notably, Cases 3–7 and Case 9 reported in the literature also had a history of mild infection prior to disease onset. Actively growing children inherently possess high energy demands, and infection-induced cellular stress further elevates these metabolic requirements. However, mitochondria compromised by *PMPCB* mutations cannot meet this increased systemic energy demand. This energy deficit induces pathological changes in highly energy-dependent systems, such as the nervous system, heart, and skeletal muscles, thereby exacerbating the disease. Furthermore, previous research has demonstrated a strong correlation between the *PMPCB* gene and immune cells. Specifically, *PMPCB* expression correlates positively with CD4+ T cells, dendritic cells, memory B cells, eosinophils, and M2 macrophages, but negatively with M0 macrophages, regulatory T cells, monocytes, and naive B cells (13). These findings suggest that pediatric patients with *PMPCB* variants may harbor underlying immunological deficiencies, rendering them highly susceptible to infections. Infection, in turn, amplifies systemic energy demands, creating a vicious cycle that severely aggravates disease progression. Consequently, for patients with *PMPCB* variants, infection can represent a devastating and potentially fatal physiological insult. When such children have a fever, active measures should be taken to reduce the fever and control the infection. In case of necessity, a professional doctor should assess whether intravenous infusion of high-dose B vitamins, leucine, or other drugs targeting acute metabolic decompensation is required.

Patients P1 and P2, treated at our institution, presented with a significantly later age of onset compared to previously reported cases. The *PMPCB* variants identified in the siblings involve distinct loci compared to those in previously described pediatric cohorts. This is a dramatically different clinical presentation. One potential explanation is residual MPP activity resulting from the identified variants, which may retain partial catalytic function and delay the onset of clinical manifestations. Additionally, hypomorphic allele effects or tissue-specific differences in gene expression could mitigate the functional impact of the mutations in early life, leading to a later presentation. Further differential expression patterns, non-genetic factors and environmental influences may also clarify the biological basis of this delayed onset phenotype. Furthermore, although siblings P1 and P2 harbor identical *PMPCB* variants, they exhibit markedly different clinical phenotypes: P1 predominantly demonstrates cognitive regression, whereas P2 primarily presents with motor regression. This indicates that identical variants at the exact same locus may have different clinical phenotypes. Collectively, these findings suggest that *PMPCB* gene biallelic variants are associated with phenotypic heterogeneity. The exact mechanisms underlying clinical heterogeneity is still unclear, potential underlying mechanisms maybe including: epigenetic modifications. the potential role of modifier genes including both autosomal and X-linked factors that could contribute to sex-specific phenotypic differences. variable gene expression patterns and tissue-specific effects. stochastic effects, including mitochondrial threshold effects and random events during development which could contribute to variable disease presentation. We admit that phenotypic heterogeneity in the siblings with identical genotypes remains an outstanding question. Further experimental studies are warranted to elucidate the pathogenesis of the phenotypic variations associated with *PMPCB* variants.

Cranial MRI revealed progressive cerebellar atrophy and/or abnormal signals in the basal ganglia in all patients except P8. Given that P8 died at 6 month old, it was highly probable that the neurological imaging abnormalities had not yet manifested. This suggests that cerebellar atrophy is the primary neuroimaging hallmark associated with *PMPCB* gene variants. All 9 patients harbored *PMPCB* biallelic variants, including six with compound heterozygous variants and three with homozygous variants. By September 2025, the HGMD has documented a total of 13 *PMPCB* gene variants, specifically c.470T > G, c.523C > T, c.524G > A, c.528A > C, c.530T > G, c.601G > C, c.766G > C, c.953C > G, c.986G > A, c.1079C > G, c.1087T > C, c.1265T > C, and c.99 + 1G > C. The current literature includes only nine patients, all reported as sporadic cases, underscoring the extreme rarity of *PMPCB* variants. Furthermore, eight distinct variants were identified in this cohort of nine patients, comprising seven missense variants and one nonsense variant. This indicates that *PMPCB* alterations are predominantly missense variants.

Silencing *PMPCB* causes mitochondrial dysfunction, highlighting its crucial role in maintaining mitochondrial homeostasis (14). Among the nine pediatric patients, all except Case 9 (who experienced a relatively stable period) exhibited rapid disease progression. Notably, Case 8 Died within just three months of disease onset. The relatively mild, late-onset phenotype in Case 9 may be attributed to variable aberrant splicing of the c.1330-2A > T variant *in vivo*, which preserves residual, normally spliced *PMPCB* mRNA in tissues (12). Conversely, the severe phenotypes associated with homozygous *PMPCB* variants likely result from a more profound impairment of MPP activity, suggesting a direct correlation between phenotypic severity and the severity of MPP dysfunction (6). Knocking down either MPP subunit impaired cell viability, clonal growth, and mitochondrial activity (15). Notably, the impact of *PMPCB* knockdown was particularly pronounced, resulting in a 34% reduction in intracellular mitochondrial activity. The mitochondrial function is especially critical in the brain due to its high energy demands. Inhibiting human *PMPCB* leads to the accumulation of intact PINK1 protein, which is normally cleaved by MPP in healthy mitochondria (16). This accumulation subsequently induces PINK1-Parkin-mediated mitophagy (17) and apoptosis (18). Furthermore, *PMPCB* as a critical gene that exhibits a synthetic interaction with eIF4E (19). As a key translation initiation factor, eIF4E is essential for eukaryotic gene translation and acts as a known oncogenic driver. Depleting *PMPCB* triggers mitochondrial proteotoxic stress, which subsequently activates an eIF4E-dependent autophagic survival response. Collectively, these findings indicate that *PMPCB* deficiency triggers a cascade of deleterious cellular and systemic effects.

The DynaMut and AlphaFold structural analyses presented in this study are purely computational and provide hypothesis-generating insights only. They should not be conflated with the experimental functional data (qPCR and Western blot). Experimental validation of protein stability and folding for both variants is needed. Analysis of the GTEx database revealed that, in addition to the brain, *PMPCB* is highly expressed in lymphocytes, blood vessels, and adrenal glands. To validate the pathogenicity of the variants identified in the affected siblings, we analyzed peripheral blood samples from patient P1 and age-matched healthy controls. qPCR and Western blotting demonstrated that both *PMPCB* mRNA and PMPCB protein levels were significantly reduced in the patient compared to the control. However, nearly half of the mRNA could be detected in the patient samples, while the PMPCB protein was almost completely undetectable. The discrepancy between the residual *PMPCB* mRNA levels and the nearly undetectable protein expression may be attributed to multiple factors, including transcriptional and post-translational mechanisms. First, this may involve nonsense-mediated mRNA decay (NMD). Specifically, the transcript harboring the premature termination codon is likely targeted for degradation by the NMD machinery, thereby preventing its translation into a truncated protein. The remaining mRNA species, which carries the p.Thr360Arg missense variant, may produce a protein with reduced stability, further contributing to the diminished PMPCB protein levels observed in patient cells. Moreover, both the truncated and misfolded proteins fail to correctly assemble into functional *α*/*β* heterodimers, and the unassembled free subunits are preferentially targeted for degradation. In addition, our study was conducted on PBMCs. Given that PBMCs are rapidly dividing hematopoietic cells, their robust mitochondrial quality control and high mitochondrial turnover rate may further accelerate the clearance of aberrant PMPCB proteins, thereby potentially amplifying the protein-loss phenotype.

Furthermore, using an FXN antibody, we did not detect FXN protein in patient-derived PBMCs lysates, whereas it was detected in samples from healthy pediatric controls and 293 T cells. This likely reflects profoundly reduced FXN expression in the patient's PBMCs, falling below the assay's limit of detection. FXN is a small (210 amino acids), evolutionarily conserved mitochondrial protein encoded by the nuclear genome and localized to mitochondria. During this targeting process, the targeting sequence is removed via a two-step proteolytic cleavage to generate the mature protein (20). Specifically, MPP sequentially cleaves the FXN precursor into an intermediate form and then into the mature protein (20). Functionally, FXN is essential for the synthesis of ISCs and facilitates their assembly into mitochondrial protein complexes. Consequently, FXN deficiency leads to mitochondrial iron accumulation, extensive oxidative damage, loss of myelinated fibers, and ultimately, cell death (21). In humans, mutations that impair FXN expression or function cause Friedreich ataxia, a progressive neurodegenerative and cardiac disorder (22).

FXN is primarily expressed in tissues with high metabolic demand, such as the brain, heart, liver, and kidneys (23). It contributes to iron homeostasis and acts as a chaperone protein in the biogenesis of ISCs, which are essential cofactors for enzymatic function and oxidative phosphorylation. Under conditions of iron overload, FXN functions as an iron storage protein, exerting a crucial antioxidant effect by mitigating the generation of reactive oxygen species (ROS). Consequently, FXN downregulation induces mitochondrial dysfunction, decreased ATP production, oxidative stress, free radical accumulation, and subsequent cell death. Although FXN is highly expressed in the mitochondria of both neurons and astrocytes, its expression is significantly higher in neurons, rendering them particularly susceptible to FXN deficiency. Indeed, the complete ablation of FXN ultimately leads to neuronal death (23). FXN positively regulates fatty acid oxidation during angiotensin II-induced endothelial-to-mesenchymal transition (EndoMT) (24). Conversely, FXN deficiency accelerates this process. Furthermore, FXN knockdown exacerbates the angiotensin II-induced elevation of mitochondrial Fe2+ levels. This mitochondrial iron overload triggers the production of free radicals, which subsequently alter the mitochondrial genome and proteome via oxidative stress. Additionally, FXN inhibits cell proliferation and attenuates both colony-forming and migratory capacities (25). FXN knockdown significantly exacerbates erastin-induced cell death, characterized by cell membrane disruption, aggravated lipid peroxidation, and impaired mitochondrial morphology. In contrast, FXN overexpression maintains mitochondrial integrity, reduces free iron levels, and enhances cellular resistance to erastin-induced ferroptosis, these establish FXN as a crucial regulator of ferroptosis. Moreover, FXN deficiency induces immune metabolic dysregulation in microglia and promotes glucose catabolism, thereby amplifying pro-inflammatory response. Collectively, these findings indicate that FXN is indispensable for maintaining normal physiological function. However, in the present study, FXN was undetectable in the PBMCs of P1. Combined with their clinical manifestations, this molecular evidenc suggests that the biallelic variants identified in the siblings are likely to be pathogenic.

A male patient diagnosed with progressive episodic ataxia at age 61, whose father had also exhibited late-onset ataxia several years prior to his death (26). His brothers similarly presented with ataxia. Both whole-genome sequencing (WGS) and RNA sequencing (RNA-seq) identified an intronic variant in the *PMPCB* gene (NM_004279: c.1154 + 5G > C), which was predicted to induce exon 9 skipping. This skipping results in the loss of a substantial portion of the MPP-*β* protein, specifically two *α*-helices and two *β*-sheets (27). Consequently, *in silico* tools predicted protein misfolding and a complete loss of function of the resulting peptidase. Given that MPP-*β* is essential for processing proteins associated with neurodegeneration (namely, FXN and PINK1) (16), its alteration is highly likely to exert a detrimental effect on cerebellar function. Therefore, the authors proposed that this variant exerts a dose-dependent pathogenic effect. While a single wild-type *PMPCB* allele is sufficient to maintain basal physiological functions [30], a monoallelic loss of function may lead to a milder, late-onset dominant neurodegenerative phenotype (16). In the present study, the *PMPCB* c.1351C > T variant identified in our pediatric patient is a novel mutation. Mutation Taster predicted the c.1351C > T (p.Arg451*) variant to be “disease-causing”, as it introduces a premature stop codon that yields a truncated protein, likely triggering nonsense-mediated mRNA decay (NMD). In previously reported pediatric cohorts with *PMPCB* mutations, patients P3 to P9 all harbored compound heterozygous or homozygous variants. In patients P1 and P2, the c.1079C > G and c.1351C > T variants were inherited from their father and mother, respectively, both of whom exhibited normal phenotypes. Considering the dose-dependent pathogenic effect (12, 26), alongside the PMPCB and FXN expression profiles in the patients' peripheral blood, we postulate that these compound heterozygous variants are responsible for the phenotypes observed in P1 and P2. Therefore, the *PMPCB* c.1351C > T variant reported herein is a novel candidate pathogenic variant, may further expanding the mutational spectrum of the *PMPCB* gene.

We also considered PINK1, another MPP substrate mentioned in the above discussion. However, we did not examine PINK1 for the following reasons: First, PINK1 is typically undetectable in healthy mitochondria because it is rapidly processed by MPP and subsequently degraded by the mitochondrial protease PARL. Secondly, Reliable detection of PINK1 accumulation requires dissipation of mitochondrial membrane potential (e.g., by CCCP treatment) to stabilize full-length PINK1 on the outer mitochondrial membrane. Inducing membrane potential dissipation in patient-derived PBMCs is technically challenging in an exploratory clinical study and would require additional optimization and sample volumes that were not available from this pediatric patient. Importantly, prior MMDS6 studies did not specifically examine PINK1 either (6), and the primary phenotypic readout consistently used was FXN maturation status. We have therefore retained FXN as the most appropriate and well-supported substrate for assessing MPP dysfunction in this exploratory study, while acknowledging that PMPCB processes multiple substrates. We recognize that PMPCB/MPP processes a broad array of mitochondrial presequence-containing substrates, and that the pathogenic mechanism in MMDS6 likely involves impaired processing of multiple proteins converging on mitochondrial dysfunction — with FXN being one well-characterized and clinically relevant component, rather than the sole downstream pathway.

Specific targeted therapies for MMDS6 are currently lacking. Clinical management primarily relies on a metabolic cocktail for symptomatic relief, encompassing respiratory chain cofactors (e.g., vitamin B2 and coenzyme Q10), antioxidants (e.g., vitamin E and idebenone), and agents to correct secondary biochemical defects (e.g., levocarnitine and arginine). Prior to admission at our institution, the sbilings underwent extensive evaluations at multiple hospitals. Notably P2 was initially misdiagnosed with dystonia and prescribed levodopa/benserazide (Madopar), which was discontinued following symptom exacerbation. This prolonged diagnostic odyssey imposed a substantial financial burden on the families before genetic testing established a definitive diagnosis of pathogenic *PMPCB* variants. Ultimately, these cases underscore the critical importance of early genetic testing for pediatric patients presenting with motor and speech developmental delays or regression. Timely genetic screening can prevent misdiagnosis, streamline clinical management, and alleviate the profound economic and psychological burdens associated with a prolonged diagnostic journey.

Based on the ACMG standards and guidelines for the interpretation of sequence variants, c.1079C > G and c.1351C > T are classified as “VUS” because of insufficient evidence(PM2_Supporting + PP3). However, based on the clinical phenotypes of the siblings and our *in vitro* results on its impact on the expression levels of the *PMPCB* gene and FXN protein, these variations can be classified as potentially pathogenic. We conclude that these mutations are candidate pathogenic variants pending functional validation for the disease of the proband, who were accordingly diagnosed with MMDS6.

In conclusion, this study first describes novel pathogenic variants associated with MMDS6, providing molecular evidence for the pathogenicity of c.1079C > G and c.1351C > T, summarizes the 7 molecularly proven families reported to date, and expands the clinical and variant spectrum of the syndrome. Our results emphasize the importance of genetic testing of patients with MMDS6.Furthermore, this research provides preliminary insights into the molecular mechanisms underlying *PMPCB* variant-induced pathogenesis. We preliminary speculate that *PMPCB* might be a critical upstream regulator of FXN maturation.

However, several limitations of this study should be acknowledged. First, owing to the invasive nature of brain tissue sampling, peripheral blood was utilized as an accessible surrogate specimen. Consequently, this surrogate tissue may not accurately recapitulate the phenotypic changes occurring within neural cells. Secondly, although P1 and P2 carry the same variant, their clinical manifestations are not entirely consistent. This suggests that molecular findings may differ between P1 and P2. However, due to constraints such as experimental costs, we have only investigated changes in peripheral blood-related indices from P1 in the present study. Future investigations utilizing neuron-specific models and enrolling larger sample cohorts are warranted to comprehensively elucidate the pathogenic mechanisms underlying *PMPCB* variants.

## Data Availability

The original genetic datasets generated in this study are not publicly available due to ethical and privacy restrictions to protect participant confidentiality. The datasets can be obtained from the corresponding author upon reasonable written request, which will be subject to institutional ethical review and signing of a data access agreement. All other data is included in the article/Supplementary Material.

## Supplementary material

Supplementary Video 1 Video of Patient P1 Walking.
