# Supplementary material for: Clinical and genetic analysis of multiple mitochondrial dysfunctions syndrome type 6 caused by biallelic PMPCB variants in children
Source: Front Pediatr. 2026 Sep 23;14:1827306. doi: 10.3389/fped.2026.1827306 (PMC13642847; doi:10.3389/fped.2026.1827306)
Supplement: Supplementary file 1 [file Table1.docx]

Variant Filtering Methods:

1. Low-Quality Filtering

1.1 Strand Bias: Remove variants with strand bias > 95%

1.2 Site Depth: Remove sites with a read depth less than 8

1.3 Site Heterozygosity: Remove sites with heterozygosity less than 15% (whitelist rescue available)

1.4 Site Quality: Remove sites with an average quality score less than 17

2. High-Frequency Variant Filtering by Population Frequency

2.1 Filter high-frequency variants with a gnomAD database allele frequency above 2% (whitelist rescue available)

2.2 Filter high-frequency variants from the internal database (sequencing platform artifacts / false-positive sites)
